# Social cognition and interactive training for first-episode, drug-naïve MDD patients

**DOI:** 10.3389/fpsyt.2025.1566811

**Published:** 2025-10-15

**Authors:** Jiaxin Li, Ru Li, Dazhi Li, Shiyi Suzy Ji, Xingguang Luo, Yong Zhang

**Affiliations:** ^1^ Unit of Bipolar Disorder, Tianjin Anding Hospital, Tianjin, China; ^2^ Department of Counseling and Clinical Psychology, Teachers College, Columbia University, New York, NY, United States; ^3^ Department of Psychiatry, Yale University School of Medicine, New Haven, CT, United States

**Keywords:** BDNF, GDNF, MDD, psychosocial function, SCIT

## Abstract

**Objective:**

This study aimed to explore the impacts of social cognition and interaction training (SCIT) on serum brain-derived neurotrophic factor (BDNF), glial cell line-derived neurotrophic factor (GDNF) levels, and psychosocial function in first-episode, drug-naïve (FEDN) major depressive disorder (MDD) patients.

**Methods:**

In this 8-week randomized controlled trial (RCT), 45 MDD patients were assigned to SCIT group and 39 to cognitive behavioral therapy (CBT) group. The 17-item Hamilton Rating Scale (HDRS-17) and the Functioning Assessment Short Test (FAST) were performed to measure depressive symptoms and functional impairment severity, respectively. We also collected blood samples for serum BDNF/GDNF level detection.

**Results:**

Compared to CBT, SCIT demonstrated significantly greater improvements in total FAST scores (*F* (1,82) = 109.21, *p* < 0.001, $\eta_{p}^{2}\;=\;0.57$ ); especially in occupational (*F* (1,82) = 16.69, *p* < 0.001, $\eta_{p}^{2}=0.17$ ); cognitive (*F* (1,82) = 103.51, *p* < 0.001, $\eta_{p}^{2}=0.56$ ), and interpersonal relationship domains (*F* (1,82) =65.07, *p* < 0.001, $\eta_{p}^{2}=0.44$ ). Changes in serum GDNF levels were positively associated with changes in autonomy (*r* (40) = 0.32, 95% *CI =* [0.02, 0.57], *p* = 0.038), and financial domains (*r* (40) = 0.44, 95% *CI =* [0.15, 0.65], *p* = 0.004) in SCIT group.

**Conclusion:**

Improvements in social function through SCIT can be effectively generalized to MDD patients. Moreover, improved GDNF levels were associated with improvements in specific aspects of social functioning post-SCIT.

## 1 Introduction

Major depressive disorder (MDD) is a prevalent illness, with 12-month and lifetime prevalences of 1.6% and 1.8% in China (1). MDD has a deleterious effect on patients’ daily functioning in various aspects, including but not limited to personal relationships, social life, and vocational efficacy, which significantly impairs psychosocial functioning and diminishes the quality of life (QoL) (2–4). Almost 70% of MDD patients do not achieve long-term functional recovery post-first-line antidepressant intervention (5). Research indicates that the risk of recurrence increases by about 12% for every 1-point increase in functional impairment scores (6). The World Health Organization has projected that depression will become the leading disease burden in high-income countries by 2030. Notably, psychosocial dysfunction represents a pronounced indirect cost to the burden of disease (7). Previous studies have demonstrated that cognitive deficits persist even after improvement and remission of mood symptoms, mediating the relationship between psychosocial deficits and MDD, leading to poor efficacy and disease recurrence (6–8). There is an urgent need to explore clinical interventions that can improve the social functioning of MDD patients (3).

Brain-derived neurotrophic factor (BDNF) and glial cell line-derived neurotrophic factor (GDNF) participate in specific neuronal growth, differentiation, and synaptic plasticity (9). Of note, accumulated evidence has summarized that BDNF/GDNF might constitute a neurobiological substrate of cognitive dysfunction in neuropsychiatric disorders (10, 11). So far, correlations between social function/cognition impairment and/or BDNF/GDNF levels have been reported in anorexia nervosa, schizophrenia, and obsessive-compulsive disorder (OCD) patients (12–14). It appears to be a trend suggesting that higher levels of BDNF/GDNF are associated with better social cognition and/or social functioning in patients with mental illnesses. Unfortunately, when exploring the association between cognitive intervention and changes in BDNF and GDNF concentrations in the MDD population, da Silva et al. failed (15). Psychosocial dysfunction may act as a secondary consequence of cognitive impairment, and both may play a critical role in the prognosis of depression (7, 8). Thus, it is helpful to explore the correlation between social function/cognition and BDNF/GDNF levels, especially for first-episode, drug-naïve (FEDN) MDD patients.

Social cognition and interaction training (SCIT) was primarily developed to improve social functioning through social cognition in schizophrenia individuals, which is feasible and effective in improving QoL, emotion recognition, and social skills in patients with schizophrenia and its spectrum disorders (16). In bipolar disorder (BD) population, SCIT has produced greater improvements in occupational and interpersonal function, as well as in visual-verbal cognitive abilities, compared to psychoeducation interventions (17). In addition, by targeting social cognition, a 14-week SCIT intervention has been proven to improve MDD patients’ emotional perception, theory of mind, and attributional styles (18). Social function, as an urgent problem that needs to be addressed, requires prompt attention (3). Interpersonal and occupational declines, as important components of social functioning, persist even when depression is remitted, posing high-risk factors for MDD recurrence and relapse, and adversely affecting the QoL of MDD patients (19, 20). Given the relationship between social function and social cognition (7), SCIT’s role in social functioning in MDD patients needs further discussion.

Cognitive-behavioral therapy (CBT) has been widely applied to improve depressive symptoms and cognitive deficits in the MDD population (21), while its role in improving social function across various mental illnesses (e.g., bipolar disorder, social anxiety disorder, chronic depression) has also been proven (22–24). It is recognized that both psychotherapy and pharmacotherapy are biological treatments that affect the brain both in similar and different ways (25). The value of neurotrophins in the prognosis of antidepressant treatment has been discussed (11). However, biomarkers capable of predicting the outcomes of psychotherapy remain elusive. As mediators of neuroplasticity, BDNF/GDNF represent promising targets for research in this field. Although BDNF might act as a promising genetic marker for treatment response to CBT intervention in depression has been discussed, BDNF levels post-CBT treatment remain controversial (26, 27). Unfortunately, no study has paid attention to the impacts of SCIT on improving social functioning in FEDN MDD patients, and BDNF or GDNF levels post-SCIT are still unknown (18). Taken together, the investigation of changes in neurotrophic factors following psychotherapy holds significant clinical relevance.

Therefore, our main purpose was to discover whether SCIT intervention is more likely to improve social function and GDNF/BDNF levels compared to CBT intervention, and to explore whether the improvement of FAST including its six domains is related to biomarkers GDNF and BDNF in FEDN MDD patients.

## 2 Methods and materials

### 2.1 Subjects

This study was performed at the Tianjin Anding Hospital from December 2018 to June 2019. Ninety outpatients with FEDN MDD aged 18–60 years were enrolled according to the Diagnostic and Statistical Manual of Mental Disorders, 5th ed (DSM-5), with a baseline score ≥ 18 on the 17-item Hamilton Rating Scale (HDRS-17). Patients with severe medical conditions, substance abuse disorders, and other severe mental illnesses, i.e., schizophrenia, BD, mental retardation, etc., were excluded. Additionally, patients who received any regular psychotherapy, such as CBT or psychoeducation before enrollment were also ruled out. The study was conducted according to the Declaration of Helsinki. This study was approved by the Ethics Committee of the Tianjin Anding Hospital (Ethics number: 2018-020). Besides, written informed consent was obtained from all participants. This study was registered on http://www.chictr.org.cn/(Identifier number: ChiCTRIIR-17010453).

### 2.2 Procedures

To ensure allocation concealment, the sequentially numbered, opaque, sealed envelope (SNOSE) technique was employed. The envelopes were prepared by people who was not involved in participant recruitment or intervention. After a participant completed the baseline assessment, the psychiatrists would open the next sequentially numbered envelope to reveal the group assignment: SCIT or CBT. Both groups received weekly interventions for a total of 8 weeks, with each training session lasting 1.5 hours. Both at baseline and week 8, all participants were assessed by one experienced psychiatrist who was blind to any intervention conditions. Two psychiatrists who were responsible for SCIT and CBT intervention respectively were also blind to each other’s intervention, and they had to receive strict training provided by senior psychologists.

#### 2.2.1 SCIT intervention

SCIT is a manualized intervention program designed to improve psychiatric patients’ social cognitive dysfunction (28). Chan et al. translated and condensed the original SCIT protocol from a 15- to a 9-week program, which confirmed suitable for Chinese adults (29). We further slightly modified it into an 8-week program by removing the general introduction but integrating its key components into the beginning of the subsequent core sessions. Our previous study has evidenced that modified SCIT programs are effective in improving the total functioning in BD patients (17). Our eight-week training sessions were divided into three phases, to be specific, 3 sessions for understanding emotions, 3 sessions for social cognitive biases, and 2 sessions for operating integration practically. To maintain the quality of the intervention, two therapists accepted the training program of the SCIT by Dr. Chan and passed the training trial.

#### 2.2.2 CBT intervention

The handbook of CBT was structured in agreement with Aaron Beck’s theory (30). According to this model, distorted or dysfunctional thinking—which adversely affects mood and behavior— is common to all psychological disorders. An accurate assessment and modification in thinking is associated with improvements in mood and behavior. Lasting improvements are achieved through the modification of the basic dysfunctional beliefs. Its early effectiveness has been verified in FEDN MDD patients (21). FEDN MDD patients in the CBT group received equal-time training, sessions focused on cognitive reconstruction (3 sessions), emotional transformation (3 sessions), and behavioral training (2 sessions).

#### 2.2.3 Pharmacological interventions

Selective serotonin reuptake inhibitors (SSRIs) or serotonin-norepinephrine reuptake inhibitors (SNRIs) with flexible doses were allowed to relieve depressive symptoms, including sertraline, fluoxetine, escitalopram or citalopram, duloxetine and venlafaxine. Non-benzodiazepines such as zopiclone (7.5 mg/day) and zolpidem (5–10 mg/day) were used to improve insomnia, but benzodiazepine sedative-hypnotics were avoided due to their potential for inducing cognitive impairment.

### 2.3 Materials

#### 2.3.1 Demographic information

Baseline sociodemographic characteristics of enrolled patients, including age, gender, education years, marriage, duration of MDD, and family history were collected.

#### 2.3.2 Depressive symptoms assessment

The HDRS-17 (31) was performed to measure depressive severity. Most items are rated on a 5-point scale of 0-4, and a small number of items are rated on a 3-point scale of 0-2. The more severe the depressive symptoms, the higher the score.

#### 2.3.3 Social function assessment

The Functioning Assessment Short Test (FAST) was used to measure functional impairment across six domains: autonomy, occupational functioning, cognitive functioning, financial issues, interpersonal relationship, and leisure time. It consists of 24 items rated on a 4-point scale ranging from 0 to 3, with a higher total score indicating more severe functional impairment (32). The FAST–Chinese version has been verified for good validity and reliability in BD patients (33). Additionally, the FAST scale has been validated for use with individuals experiencing depression, providing a detailed assessment of daily functioning (34, 35).

#### 2.3.4 Serum BDNF and GDNF concentration

We collected 10 ml of blood samples to detect serum BDNF and GDNF levels both at baseline and week 8 for all participants. Venous blood was collected without anticoagulant between 7 and 8 a.m. and immediately sent to the laboratory for serum preparation. Within 1 hour of collection, blood samples were separated into two parts for BDNF and GDNF testing to minimize variability. Serum samples were separated by centrifugation post 1 hour of incubation and stored at − 80°C for further analysis. The blood samples were analyzed by a commercial ELISA kit (Promega. USA), using the protocol already depicted (36). The intra- and inter-assay coefficients of variation were < 4% and < 5%, respectively. Importantly, lab personnel were blinded to group allocation during ELISA testing.

### 2.4 Statistical analysis

SPSS 26.0 was used to analyze the statistical data, and besides, GPower 3.1 was used to calculate the sample size and verify the statistical power, while GraphPad Prism 9.0 was used to plot graphs. The characteristics of sociodemographic and clinical-related variables (HDRS, FAST including its six domains, and serum BDNF/GDNF levels) between SCIT and CBT groups were analyzed using independent sample *t*-tests for continuous variables and chi-square tests for categorical variables. Repeated-measures analyses of variances (ANOVA) were performed to detect the effects of the two different interventions on scores of HDRS, FAST including its six domains, as well as serum levels of BDNF and GDNF from pre- to post-treatment. Perform pairwise comparisons on variables with significant intervention * time interaction, all of which have been corrected by Bonferroni or Sidak. Pearson correlations were performed to explore the association between changes in the FAST scale and serum levels of BDNF and/or GDNF both in SCIT and CBT groups. All statistical tests were two-tailed (*p* < 0.05 is considered significant).

## 3 Results

### 3.1 Demographic and clinical characteristics of all participants at baseline

According to Gpower 3.1, a medium level effect size (*f* = 0.25) (see Cohen’s criteria) was set, with an alpha (α) level set at 0.05 (two-tailed) and a desired power (1-β) of 0.80. A total sample size of N = 34 resulted in a 0.81 actual power. Actually, ninety FEDN MDD patients were recruited at baseline. According to the per-protocol analysis principle, 6 patients (4 for SCIT group, 2 for CBT group) were excluded because they uncompleted all assessments during 8 consecutive weeks. There were no differences in demographics, HDRS, FAST including its six domains, and serum BDNF/GDNF levels between excluded and included patients (all *p’s* > 0.05). Finally, 84 FEDN MDD patients completed the interventions and all assessments during the 8-week follow-up, including 45 patients for SCIT group and 39 patients for CBT group. There was no significant difference in gender (*χ^2^* = 0.02,*φ* = 0.02, *p* = 0.886), age (*t* = 0.64, Cohen’s *d* = 0.14, *p* = 0.524), education years (*t* = -0.38, Cohen’s *d* = 0.08, *p* = 0.709), marriage status (*χ^2^* = 2.35, Cramér’s*V* = 0.17, *p* = 0.308), the current duration (*t* = -1.08, Cohen’s *d* = 0.24, *p* = 0.283), and family history (*χ^2^* = 2.76, *φ* = 0.18, *p* = 0.096) between SCIT group and CBT group, see **Table 1**. The type of antidepressants according to flexible dosage was also not significantly different between groups (*χ^2^* = 0.44, Cramér’s*V* = 0.07, *p* = 0.994). Moreover, we compared the scores of HDRS, FAST including its six domains, as well as serum BDNF and GDNF levels between groups using baseline data, with results revealing no statistical differences in clinical-related variables (all *p’s* > 0.05), see **Table 1**.

**Table 1:** Demographic and clinical characteristics between SCIT group and CBT group at baseline.

Variables	SCIT (*n* = 45)	CBT (*n* = 39)	*T/χ2*	Effect size	95%CI for^1 2 3^	*P* value
Age (years)^† 1^	34.9(12.3)	36.6(12.7)	0.64	0.14	[-0.28,0.56]	0.524
Gender (Female)^# 2^	27.0(60.0)	24.0(61.5)	0.02	0.02	[-0.22,0.19]	0.886
Marriage^# 3^			2.35	0.17	[0.00,0.35]	0.308
single	23.0(51.1)	14.0(35.9)	——			——
married	19.0(42.2)	23.0(59.0)	——			——
divorced	3.0(6.7)	2.0(5.1)	——			——
Family history (yes)^# 2^	7.0(15.6)	12.0(30.8)	2.76	0.18	[-0.38,0.02]	0.096
Education (years)^† 1^	13.7(2.7)	13.5(2.9)	-0.38	0.08	[-0.50,0.34]	0.709
duration of MDD (months)^† 1^	14.0(15.4)	10.8(11.6)	-1.08	0.24	[-0.66,0.20]	0.283
HDRS^† 1^	27.6(6.0)	26.3(4.9)	-1.06	0.23	[-0.65,0.20]	0.291
BDNF (ng/ml)^† 1^	40.3(4.8)	40.3(5.6)	-0.01	0.00	[-0.42,0.42]	0.994
GDNF (ng/ml)^† 1^	377.2(65.2)	373.0(68.3)	-0.29	0.06	[-0.48,0.36]	0.771
FAST total score^† 1^	26.2(10.3)	26.5(11.4)	0.13	0.03	[-0.39,0.45]	0.895
- Autonomy^† 1^	3.0(2.1)	2.7(1.7)	-0.67	0.15	[-0.57,0.28]	0.505
- Occupational functioning^† 1^	5.1(3.9)	4.8(4.0)	-0.39	0.09	[-0.51,0.34]	0.698
- Cognitive functioning^† 1^	6.8(2.9)	7.6(3.5)	1.19	0.26	[-0.17,0.69]	0.237
- Financial issues^† 1^	1.5(1.6)	2.1(1.8)	1.55	0.34	[-0.09,0.77]	0.125
- Interpersonal relationship^† 1^	7.0(3.3)	6.9(4.5)	-0.15	0.03	[-0.45,0.39]	0.885
- Leisure time^† 1^	2.7(1.8)	2.4(1.7)	-0.91	0.20	[-0.62,0.23]	0.366

FAST, Functional Assessment Test Short Form; SCIT, social cognition and interaction training; CBT, cognitive-behavioral therapy; MDD, major depressive disorder; HDRS, 17-item Hamilton Depression Rating Scale; BDNF, brain-derived neurotrophic factor; GDNF, glial cell-derived neurotrophic factor; **^†^**, Mean (SD), *p*-value corresponds to independent samples *t*-tests; **^#^**, *n* (%), *p*-value corresponds to chi-square tests, ^1^, Effect Size (Cohen’s d); ^2^, Effect Size(φ); ^3^, Effect Size (Cramér’sV).

### 3.2 Changes of clinical-related variables from pre- to post-treatment

**Figure 1:**
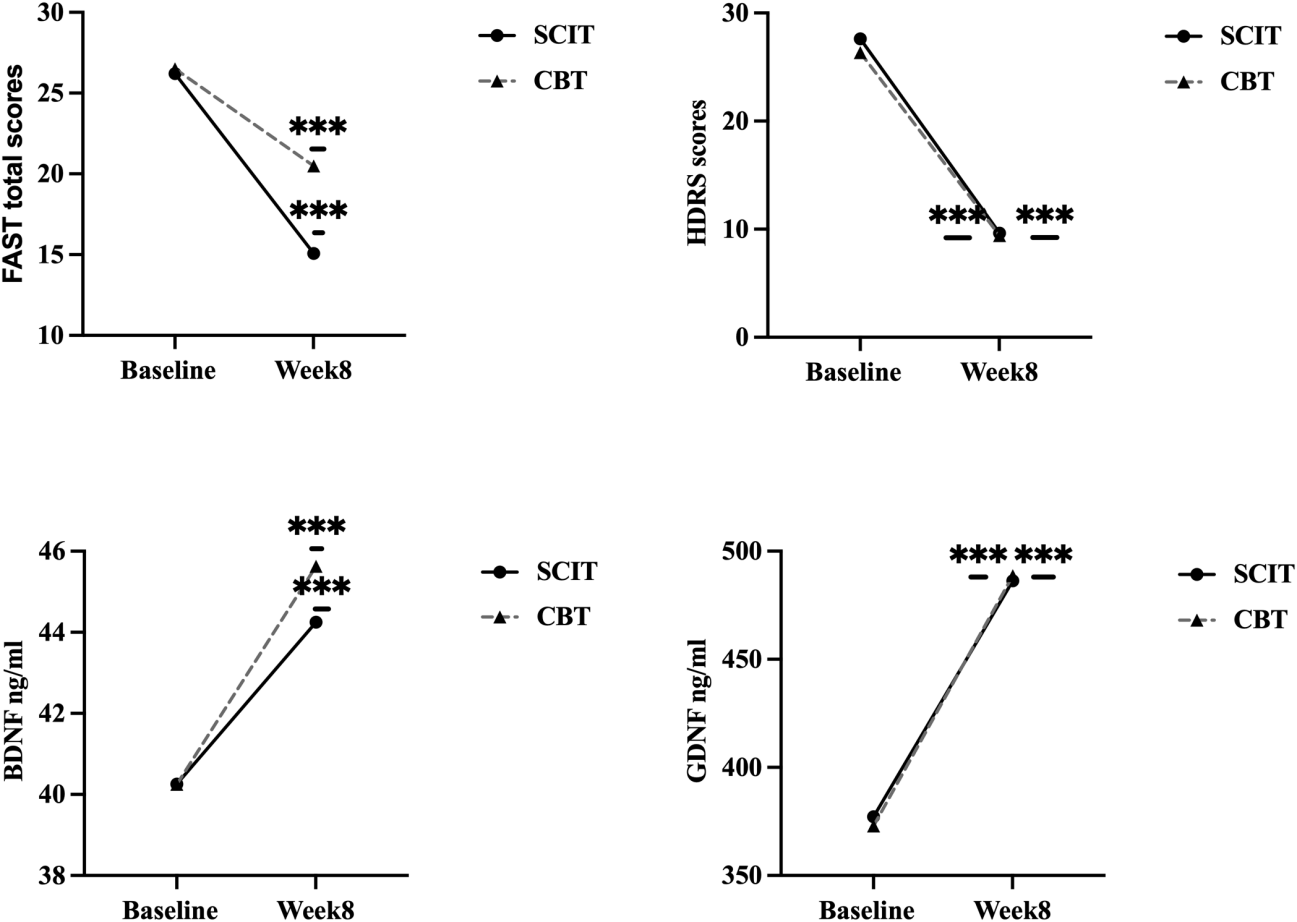
Changes of clinical-related variables from pre- to post-treatment. FAST, Functional Assessment Test Short Form; SCIT, social cognition and interaction training; CBT, cognitive-behavioral therapy; HDRS, 17-item Hamilton Depression Rating Scale; BDNF, brain-derived neurotrophic factor; GDNF, glial cell-derived neurotrophic factor; ^***^: *p* < 0.001.

**Figure 2:**
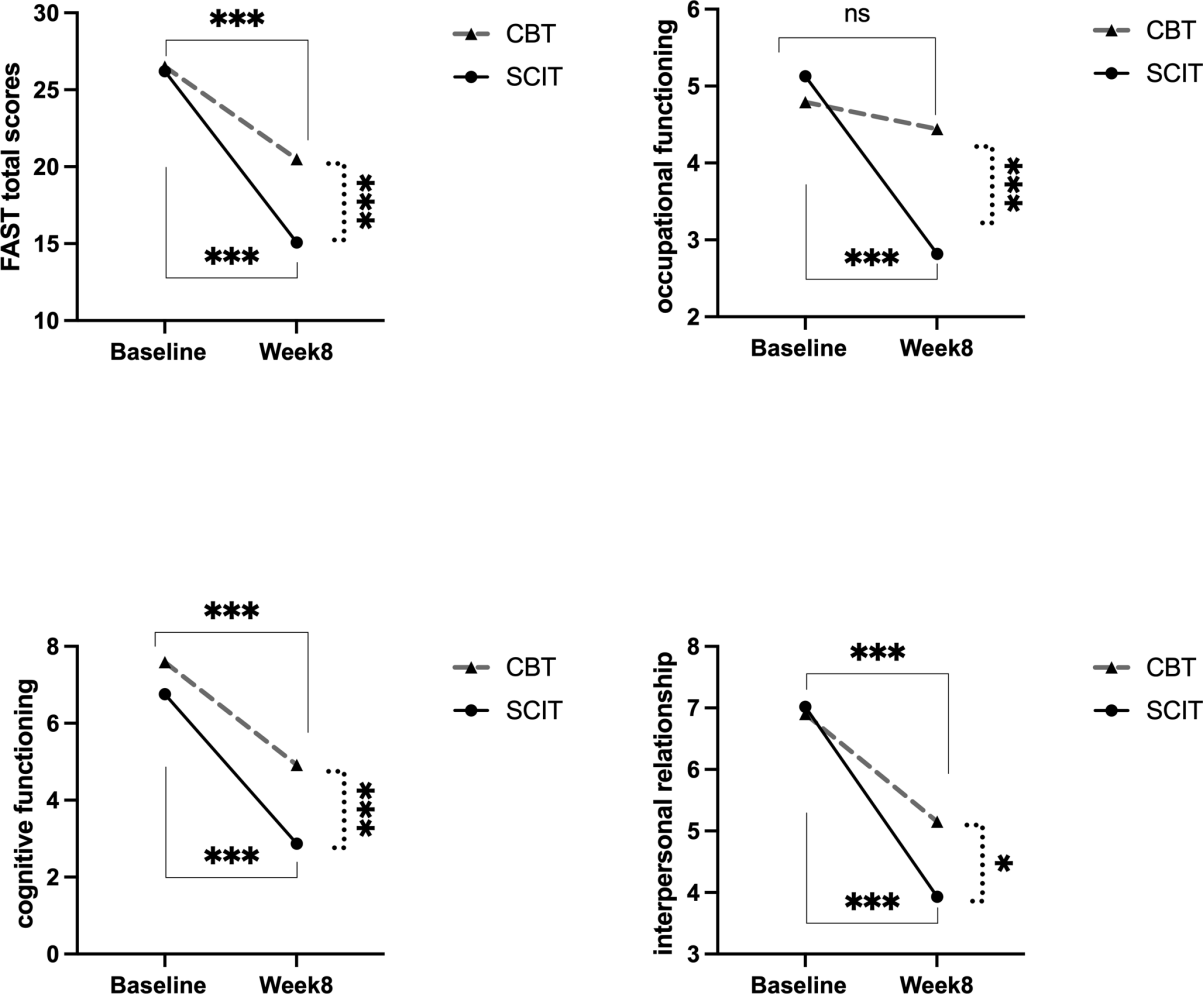
The intervention*time interaction effects on FAST and occupational, cognitive, interpersonal domains pre to post 8-week intervention in SCIT and CBT group. SCIT, social cognition and interaction training; CBT, cognitive-behavioral therapy; FAST, Functional Assessment Test Short Form; solid lines represent the time effect within groups;dashed lines indicate the between-group comparison at week 8;^***^: *p* < 0.001; ^*^: *p* < 0.05.

Repeated-measures ANOVA was performed to examine within-group, between-group, and interaction effects. Both SCIT and CBT showed a prominent improvement in FAST total scores (SCIT: *F* (1,82) = 100.78, *p* < 0.001, $\eta_{p}^{2}=0.70$ vs CBT: (1,82) = 30.70, *p* < 0.001, $\eta_{p}^{2}=0.45$ ); HDRS scores (SCIT: *F* (1,82) = 694.85 *p* < 0.001, $\eta_{p}^{2}=0.94$ vs CBT: (1,82) = 453.08, *p* < 0.001, $\eta_{p}^{2}=0.92$ ); the levels of serum BDNF (SCIT: *F* (1,82) = 17.68, *p* < 0.001, $\eta_{p}^{2}=0.29$ vs CBT: (1,82) = 19.90, *p* < 0.001, $\eta_{p}^{2}=0.34$ ) and GDNF (SCIT: *F* (1,82) = 91.80, *p* < 0.001, $\eta_{p}^{2}=0.68$ vs CBT: (1,82) = 100.91, *p* < 0.001, $\eta_{p}^{2}=0.73$ ) from pre- to post-treatment over 8 weeks, see **Figure 1**. The intervention*time interaction was significant, indicating that the two groups exhibited different trends in FAST total scores (*F* (1,82) = 10.67, *p* = 0.002, $\eta_{p}^{2}=0.12$ ); occupational functioning (*F* (1,82) = 5.53, *p* = 0.021, $\eta_{p}^{2}=0.06$ 0); cognitive functioning (*F* (1,82) = 4.75, *p* = 0.032, $\eta_{p}^{2}=0.06$ ); and interpersonal relationship of FAST(*F* (1,82) = 5.73, *p* = 0.019, $\eta_{p}^{2}=0.07$ ) over time, see **Figure 2**.

After *post-hoc* pairwise comparisons, the SCIT group showed a more pronounced decrease in FAST total scores (SCIT: *F* (1, 82) = 109.21, *p* < 0.001, $\eta_{p}^{2}=0.57$ vs CBT: *F* (1, 82) = 27.72, *p* < 0.001, $\eta_{p}^{2}=0.25$ ); occupational functioning (SCIT: *F* (1, 82) = 16.69, *p* < 0.001, $\eta_{p}^{2}=0.17$ vs CBT: *F* (1, 82) = 0.35, *p* = 0.556, $\eta_{p}^{2}=0.00$ ); cognitive functioning (SCIT: *F* (1, 82) =103.51, *p* < 0.001, $\eta_{p}^{2}=0.56$ vs CBT: *F* (1, 82) = 42.18, *p* < 0.001, $\eta_{p}^{2}=0.34$ ); and interpersonal relationship (SCIT: *F* (1, 82) = 65.07, *p* < 0.001, $\eta_{p}^{2}=0.44$ vs CBT: *F* (1, 82) =17.97, *p* < 0.001, $\eta_{p}^{2}=0.18$ ) of FAST than the CBT group. There were no differences between the two groups in the above variables at baseline (all *p’s* > 0.05) after pairwise comparisons. However, at Week 8, the SCIT group’s FAST total scores (*F* (1, 82) = 24.38, *p* < 0.001, $\eta_{p}^{2}=0.23$ ); occupational functioning (*F* (1, 82) = 23.96, *p* < 0.001, $\eta_{p}^{2}=0.23$ ); cognitive functioning (*F* (1,82) = 46.45, *p* < 0.001, $\eta_{p}^{2}=0.36$ ); and interpersonal relationship (*F* (1, 82) = 6.79, *p* = 0.011, $\eta_{p}^{2}=0.08$ ) of FAST was significantly lower than that of the CBT group, see **Figure 2**. All pairwise comparisons were Bonferroni/Sidak-corrected. There were no different trends and statistical differences in HDRS scores, BDNF, and GDNF levels between the two groups from pre- to post-intervention over 8 weeks (all *p’s* > 0.05).

### 3.3 Associations of FAST and serum BDNF/GDNF levels

**Figure 3:**
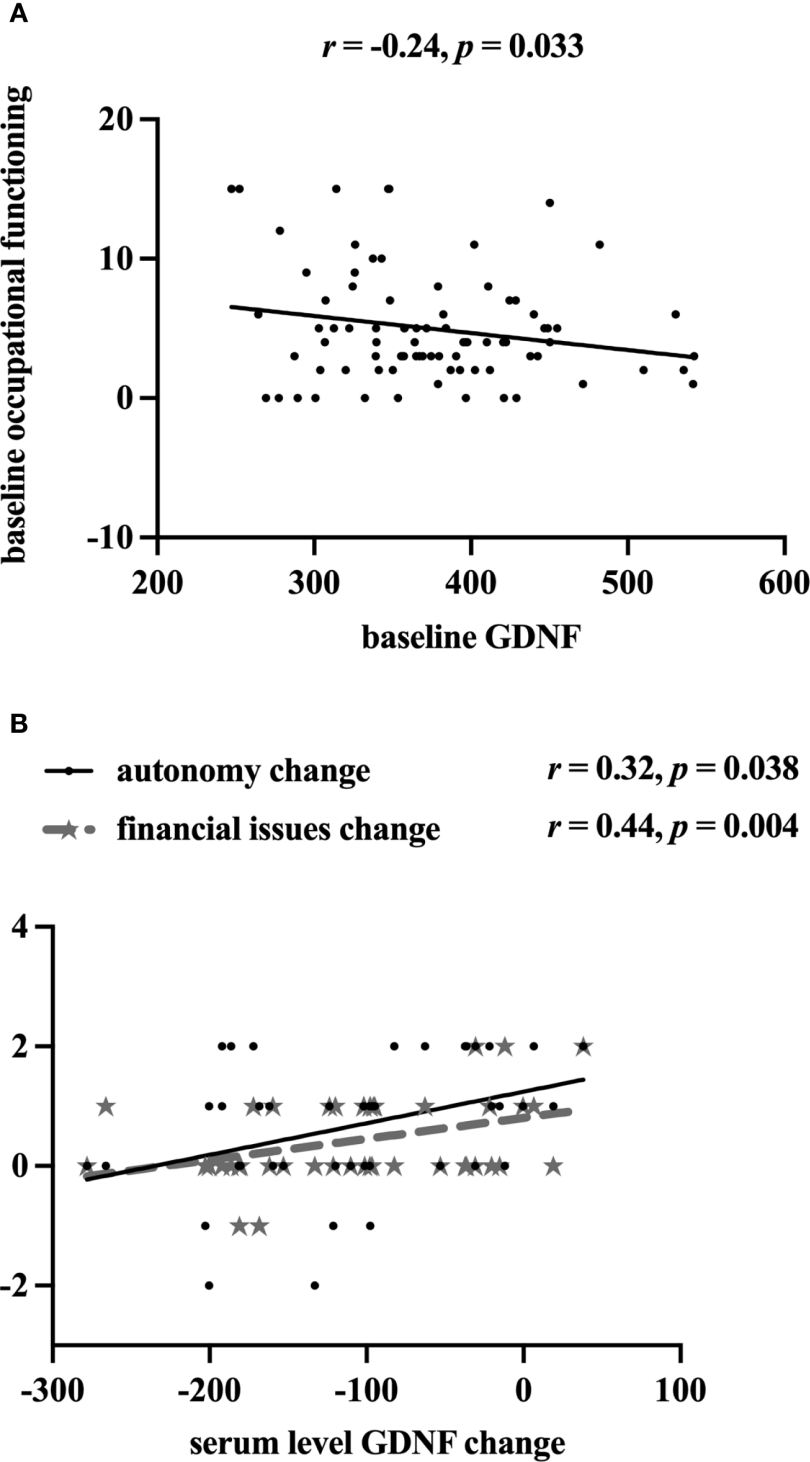
Associations between domains of FAST and serum levels of BDNF/GDNF from pre- to post-intervention. GDNF, glial cell-derived neurotrophic factor; **(A)** contains all samples at baseline, **(B)** contains only SCIT group samples from pre- to post-intervention.

Using baseline data, after controlling for age, educational years, and duration of MDD, partial correlation analysis yielded that occupational functioning was negatively associated with serum GDNF levels (*r* (79) = -0.24, 95% *CI =* [–0.43, –0.02]*, p* = 0.033) in all FEDN MDD patients, see **Figure3A**. Further analyses based on intervention measures revealed that changes in serum GDNF levels were significantly associated with changes in domains of autonomy (*r* (40) = 0.32, 95% *CI =* [0.02, 0.57], *p* = 0.038), and financial issues (*r* (40) = 0.44, 95% *CI =* [0.15, 0.65], *p* = 0.004) in SCIT group, see **Figure 3B**. To account for multiple comparisons across the examined serum biomarkers and social functioning, false discovery rate (FDR) correction was applied using the Benjamini–Hochberg procedure, significant results were considered at an FDR-adjusted *p*-value threshold of < 0.05. However, after correction, only the correlation between GDNF difference and financial issues difference still holds statistical significance (FDR-adjusted *p* = 0.49). Besides, there was no correlation between changes in FAST (including its six domains) and changes in serum GDNF or BDNF level were found in CBT group.

## 4 Discussion

This was an 8-week randomized controlled trial (RCT), which aimed to examine the impacts of SCIT on improvements in psychosocial function, depressive symptoms, and serum BDNF/GDNF levels in FEDN MDD patients. Our findings indicated that both SCIT and CBT interventions could significantly enhance scores of HDRS and total FAST, and levels of serum BDNF and GDNF in FEDN MDD patients. Notably, the SCIT group demonstrated more substantial improvements in overall social functionality, especially in interpersonal relationship, occupational capacity, and cognitive function domains compared to the CBT group. Intriguingly, enhancements in serum GDNF levels following SCIT intervention were correlated with changes in autonomy and financial issue domains of FAST. These findings support our initial hypotheses, highlighting the superior effectiveness of SCIT intervention in improving social function compared to CBT intervention. Furthermore, although preliminary exploration, our study appears to establish a potential link between elevated levels of the serum biomarker GDNF and improvements in specific aspects of social functioning.

The current research found that both SCIT and CBT exerted a positive effect on social functioning. Besides, our findings revealed that CBT had a significant improvement in depressive symptoms, demonstrating its strong efficacy in the enhancement of emotional regulation. SCIT’s role in relieving depressive symptoms in schizophrenia and BD populations has been discussed (17, 37), and we verified improved depressive symptoms in MDD population. However, our study did not find any differences in depression improvement between SCIT and CBT intervention. CBT’s role in improving depression has been verified (38). Our findings can be interpreted as a positive outcome, demonstrating that SCIT is an effective therapy to alleviate depressive symptoms that is no less effective than CBT. It is possibly due to the fact that both interventions could improve cognitive dysfunction and, in turn, depressed mood and social function. SCIT was initially designed to improve social cognitive dysfunction, and its role in schizophrenia, BD, and MDD patients has been proven (16–18, 28). In light of the association between cognitive dysfunction and subsequent functional deficits in everyday life has been explored in mental illnesses (39), there appears to be a trend that social functioning will be improved post-SCIT, and this viewpoint has been validated in schizophrenia and BD patients (16, 17). Our findings further substantiate this evidence in the MDD population. Thus, we hypothesize that SCIT could improve social cognitive capacities to support social function measured by FAST in the MDD population. However, this hypothesis needs to be validated with further cognitive function measurement.

Although previous studies have confirmed that CBT intervention could improve cognitive impairment and social dysfunction (23, 40). A strength in our findings is that SCIT has been shown to be superior to CBT in enhancing interpersonal relationship, occupational function, and cognitive function in FEDN MDD patients. In short, our research extends Zhu et al’s findings that SCIT could improve interpersonal and occupational capacities among depressed individuals (18). Interestingly, our previous study elaborated that compared to psychoeducation, SCIT exhibited greater improvements in the aforementioned domains in the BD group (11). Taken together, it appeared that SCIT benefits interpersonal and occupational function in mental illness patients. A cross-sectional analysis has proved that interpersonal interaction could reverse the effects of functional disabilities on the QoL for serious mental illness, highlighting the important role of interpersonal relationship in social function (41). Another study focused on work-targeted psychotherapy proved to remediate occupational impairment (42). We considered that SCIT not only targets to promote interpersonal interactions by ameliorating judgmental ambiguities and difficulties in work and social settings (28, 33), but also encourages patients to interact with other members to learn more social skills, which may obviously promote interpersonal activity, further boost the work skills and cognitive abilities, while CBT mainly focuses on automatic and maladaptive thought patterns (40).

To date, BDNF levels in MDD patients following psychotherapy remain controversial (26). Distinct from da Silva et al.’ finding that no changes in neurotrophic concentrations were found after psychotherapy, our study showed that both SCIT and CBT intervention can elevate serum BDNF and GDNF levels in MDD patients (15). Zhou et al. confirmed that CBT intervention could restore aberrant dynamic functional connectivity within the dorsolateral frontal anterior cortex, which may play a pivotal role in regulating both emotion and cognition in MDD patients (40). Intriguingly, BDNF and GDNF are associated with emotion and cognition, which might possess a remarkable capability to repair brain damage (9, 43). Taken together, BDNF/GDNF could be indicators of the efficacy of CBT (26). However, the present study did not find a difference between CBT and SCIT intervention in the extent of improvement in BDNF and GDNF levels, suggesting psychotherapy may have a similar mechanism of action, or that an 8-week intervention was too short to observe such variability (26, 44). Besides, the smaller sample size means that though there may be subtle differences between SCIT and CBT, this study was unlikely to identify them. Thus, our findings indicated that larger-scale, longer-term intervention should be considered to explore differences in biomarkers and functioning between SCIT and CBT intervention.

Using baseline data, we also found that serum GDNF levels exhibited a negative correlation with the impairment of occupational function. Although the association is no longer significant after FDR correction, it still suggests a trend that lower serum GDNF levels might be a risk factor for psychosocial dysfunction in MDD patients. Similarly, Okuno et al. proved that social adaptation positively correlates with plasma BDNF levels in healthy controls (45). Another study suggests that a healthy population carrying the BDNF Val Val genotype might show a better ability for social interactions (46), which could facilitate work ability (47). Accumulated human and animal experiments have shown that increased levels of BDNF and GDNF were associated with better cognitive function, including but not limited to learning and memory domains (10, 48, 49). Considering that functional disability can be a result of cognitive dysfunction (7, 8), it can be inferred that the long-term effects of higher BDNF and GDNF concentrations are advantageous for enhancing social functioning. However, we failed to replicate the correlation between BDNF and social function in the MDD population. The small sample size and eight-week duration of the study may have weakened this correlation. Moreover, this separation may provide a novel insight, suggesting that BDNF and GDNF may play distinct roles in functional recovery from MDD.

Taking SCIT intervention into further consideration, changes in serum GDNF levels correlated with improved autonomy and financial ability, with the financial aspect remaining statistically significant even after FDR correction. Unfortunately, we found no correlations between changes in BDNF and FAST including its six domains post-SCIT. The supplementary motor area is a crucial area for linking cognition to action (50), and its abnormal neural activities correlated with cognitive functioning and financial issue ability have been validated in FEDN MDD patients (51). It raises a question about whether SCIT like CBT, can act on specific brain regions, promote the secretion of GDNF, and subsequently lead to a series of social cognitive and functional changes, as we hypothesized. This inference needs to be further confirmed by future research on brain imaging combined with neurocognitive. However, we could not find these correlations in the CBT group, even though recent systematic reviews and meta-analyses have emphasized the effects of CBT on decreased dorsal anterior cingulate cortex activity which mainly involved in emotional and cognitive functions (52, 53). So far, the association between biomarkers and social function has received less attention, our study preliminarily shows a tendency that higher serum GDNF levels might be related to better social function.

Some limitations of our research should be stated. Firstly, although flexible doses of antidepressants were more suitable for clinical situations, they also introduce confounding effects that may obscure the isolated impact of psychosocial interventions. Conducting only different psychological interventions in the future will make our conclusion more convincing. Besides, enrolling a control group (e.g., waiting list, TAU) would benefit the interpretation of natural symptom trajectories. Secondly, the absence of social cognitive assessments may limit the explanation for how SCIT enhances social functioning. Previous research has suggested that SCIT might exert its positive effects on social functioning by modifying theory of mind abilities (54). Thirdly, the limited sample size, the current 8-week intervention, and absence of follow-up data limit understanding of the long-term effects of SCIT and CBT (55, 56). Lastly, the assessment was limited to the FAST scale, which may not fully capture social functioning domains in MDD patients. Future studies could incorporate broader instruments, such as the Social Disability Screening Schedule (SDSS), to explore a wider range of correlations. Considering future large-scale, long-term interventions, combined with social cognition and brain imaging techniques such as fMRI, to more precisely detect the similarities and differences in neural networks and molecular levels under the two therapies.

## 5 Conclusion

Improvements in social function through SCIT can be effectively generalized to MDD patients. Moreover, improved GDNF levels were associated with improvements in specific aspects of social functioning post-SCIT.

## Data Availability

The raw data supporting the conclusions of this article will be made available by the authors, without undue reservation.
